# A microbial flora with superior pollutant removal efficiency and its fermentation process optimization

**DOI:** 10.1186/s13568-023-01604-0

**Published:** 2023-10-17

**Authors:** Yonghong Li, Xiuxiu Wu, Yun Wang, Yingman Gao, Keke Li

**Affiliations:** 1 School of Pharmaceutical Sciences, Zhengzhou University, Zhengzhou, 450001 Henan China; 2 HeNanJinBaiHe Biotechnology Co., LTD, Anyang, 450000 Henan China; 3 Key Laboratory of Advanced Drug Preparation Technologies, Ministry of Education, Zhengzhou University, Zhengzhou, 450001 Henan China

**Keywords:** Microbial flora, Pollutant degradation, Viable bacteria, Ammonia nitrogen, Chemical oxygen demand

## Abstract

Microbial flora plays an important role in microorganism-enhanced technology. The pollutant degradation ability and viable counts of these agents are crucial to guarantee their practical application. In this study, an efficient pollutant-degrading microbial flora was screened, its medium components and culture conditions were optimized, and its effect was verified in zeolite trickling filter towers. After a 24 h culture under the optimal conditions, the viable count reached 4.76 × 10^9^ cfu/mL, with the degradation rates of ammonia nitrogen (NH_4_^+^-N), nitrate nitrogen (NO_3_^−^-N), total nitrogen (TN), total phosphorus (TP), and chemical oxygen demand (COD) increased to 93.5%, 100%, 68.3%, 32.6%, and 85%, respectively. After optimizing the feeding strategy, the concentration of viable bacteria reached 5.80 × 10^9^ cfu/mL. In the application effect verification experiment, the degradation rates of NH_4_^+^-N, TN, TP, and COD in the experimental group reached 96.69%, 75.18%, 73.82%, and 90.83%, respectively, showing a significant improvement compared to the results of the control group. The main components in the control group were *Dokdonella, Brevundimonas, Alishewanella, Rhodobacter, Pseudoxanthomonas,* and *Thauera*, whereas those in the experimental group were *Dokdonella, Proteocatella, Rhodobacter, Dechlomonas,* and *Nitrospira*. *Proteocatella, Dechlomonas, and Nitrosra*, which were unique to the experimental group, are common bacteria used for nitrogen and phosphorus removal*.* This explains the difference in the sewage treatment capacity between the two groups. This study provides an alternative sewage treatment microbial flora with a reasonable production cost and high degradation efficiency for NH_4_^+^-N, TN, TP, and COD.

**Supplementary Information:**

The online version contains supplementary material available at 10.1186/s13568-023-01604-0.

## Keypoints

- A mixed-bacteria group with a high degradation rate of NH_4_^+^-N, TN, TP, and COD was screened.
- The viable cell concentration and pollutant degradation efficiency of the screened microbial flora was increased significantly by optimizing its cultivation conditions.
- Increased pollutant degradation efficiency was verified in a zeolite trickling filter tower and explained by the microbial composition of the screened microbial flora.

**Supplementary Information:**

The online version contains supplementary material available at 10.1186/s13568-023-01604-0.

## Introduction

Water is an indispensable resource (Adimalla et al. 2020). Unfortunately, with rapid industrialization and urbanization, water pollution has become increasingly serious. Excess nitrogen destroys the ecological balance of water bodies, leading to eutrophication (Chakraborty et al. 2021; Jarvie et al. 2020). This promotes algal proliferation, leading to dissolved oxygen depletion and the death of aquatic organisms(Hu et al. 2021a, b). In sewage, nitrogen is converted to foul gas and nitrate. Nitrates may cause illnesses such as methemoglobinemia and cancer (Ward et al. 2018). They may also accumulate in aquaculture animals, weaking their immune systems, and reducing their output and quality. Therefore, it is important to improve the efficiency of nitrogen treatment in sewage. Biological technology is widely used for nitrogen removal. During biological nitrogen removal, ammonia nitrogen (NH_4_^+^-N) is converted to nitrite nitrogen (NO_2_^−^-N) and nitrate nitrogen (NO_3_^−^-N) by nitrifying bacteria and then further converted to gaseous N_2_ or N_2_O by denitrifying bacteria (Liang et al. 2017; Rajta et al. 2020).

Traditional microbial treatment of sewage involves the direct addition of sludge; however, sludge is dirty and difficult to transport. Nitrifying and denitrifying bacteria are widely used as substitutes and can be used alone or in combination. Among the nitrogen-removing bacteria, nitrifying bacteria grow slowly and play an essential role (Quartaroli et al. 2019; Zhao et al. 2020). However, commercially available nitrifying and denitrifying bacteria are expensive. Therefore, there is an urgent need to develop efficient and economical microbial flora to meet growing market demand.

Scientists have made great efforts to screen effective nitrogen-removing strains, and various heterotrophic nitrifying bacteria have been isolated, including *Pseudomonas sp*., *Alcaligenes sp*., *Arthrobacter sp*., *Acinetobacter sp*., *Bacillus sp*., *Thiosphaera sp*., *Rhodococcus sp*., and *Agrobacterium sp*. (Cai et al. 2019; Gao et al. 2020). Microorganism-enhanced technology has been extensively studied. Hu et al. used *Pseudomonas sp*. HXF1 to remove NH_4_^+^-N from groundwater (Hu et al. 2021b). Supreeth reported that plants and bacteria could cooperate to strengthen the removal of NH_4_^+^-N, total phosphorus (TP), and chemical oxygen demand (COD) (Supreeth 2022). Shen used a microalgae-bacteria system to treat domestic sewage; the results showed that the removal rates of NH_4_^+^-N, TP, and COD by co-treatment were higher than those by single treatment (Shen et al. 2017). However, it is difficult for pure strains to adapt to complex sewage environments and achieve satisfactory pollutant removal. The microbial community has superiority over the pure strains in this respect. Liu et al. used *Pseudomonas geniculata* ATCC 19374 and *Bacillus cereus* EC 3 to treat sewage. The results showed that the degradation rates of NH_4_^+^-N, TN, and COD achieved by the mixture were higher than those achieved by individual strains (Liu et al. 2020).

In addition to pollutant degradation ability, production cost and price are also important factors of microbial agents. Improving the fermentation level of microbial agents is an effective method for decreasing the production costs. The traditional method involves the optimization of medium components and culture conditions. High-density fermentation using fed-batch cultures is another effective strategy. Scheel et al. reported that *Escherichia coli* high-density fermentation was achieved by fed-batch fermentation mode, and the yield of chain-length poly(3-hydroxyalkanoates) increased from 66.8% to 99.0% (Scheel et al. 2021).

In this study, an effective pollutant-degrading bacterial flora (PDBF) was screened, and its fermentation process was optimized to promote its growth and increase its pollutant-degrading ability. Additionally, the bacterial flora was used to treat the simulated sewage to evaluate its pollutant degradation efficiency comprehensively. Finally, the composition of the bacterial flora was analyzed to explain its excellent pollutant degradation ability.

Our study lays a solid foundation for the production of microbial agents with high pollutant degradation efficiencies at a low cost.

## Materials and methods

### Inoculum

Samples A, B, C and D were taken from Zhengzhou Sewage Treatment Co., Ltd. (112^o^42′-114^o^14′ E, 34^o^16′-34^o^58′ N), xinmi city Jinmen Sewage Treatment Co., Ltd. (110° 41′-113^o^14′ E, 34^o^16′-34^o^58′ N), Henan Chengsheng Sewage Treatment Co., Ltd. (113° 40′-115^o^14′ E, 34^o^16′-34^o^58′ N) and Zhengzhou Tongji Environmental Protection Engineering Co., Ltd. (110^o^42′-116^o^17′ E and 36^o^18′-36^o^58′ N) respectively. Their water sources are domestic sewage, landfill leachate, sewage from chemical industry park and sewage from food Industry Plant.

### Media

Broth medium contains 5.0 g/L NaCl, 3.0 g/L beef paste, and 10.0 g/L peptone.

Heterotrophic nitrification liquid medium (HNLM) contains 0.5 g/L (NH_4_)_2_SO_4_, 50.0 mL/L Vickers salt solution, 5.62 g/L C_4_H_4_Na_2_O_4_. 6H_2_O and 10.0 mL/L trace elements.

Trace elements contains 5.5 g/L CaCl_2_, 5.06 g/L MnCl_2_, 1.1 g/L (NH_4_)_2_MoO_4_, 1.57 g/L CuSO_4_, 50.0 g/L EDTA, 2.2 g/L ZnSO_4_, 5.0 g/L FeSO_4_ and 1.61 g/L CoCl_2_.

The composition of heterotrophic nitrification solid medium (HNSM) was the same as that of HNLM with the addition of 15.0 g/L agar.

The inorganic salt medium contains 10.297 g/L CH_3_COONa^**.**^3H_2_O, 50.0 mL/L Vickers salt solution, 1.0 g/L (NH_4_) _2_SO_4_ and 10.0 mL/L trace elements.

The Vickers salt solution comprises 0.05 g/L FeSO_4_, 0.05 g/LMnSO_4_, 5.0 g/L K_2_HPO_4_, 2.5 g/L MgSO_4_ and 2.5 g/L NaCl.

The simulated wastewater contains 500.0 mg/L glucose, 200.0 mg/L MgSO_4_,30.0 mg/L CaCl_2_, 30.0 mg/L KH_2_PO_4_, 500.0 mg/L KHCO_3_, 100.0 mg/L NaNO_3_, 30.0 mg/L (NH_4_)_2_SO_4_ and 100.0 mg FeCl_3_.

All above-mentioned solutions were adjusted to pH 7.0 and sterilized for 20 min at 121 °C.

### Screening of PDBFs

Four samples, A, B, C, and D, were diluted 10 folds with 0.9% physiological saline and then shaken with glass beads for 1 h. Microorganisms in the samples were activated and acclimated by culturing for 24 h at 30 °C and 150 r/min in the broth medium and HNLM successively. The domesticated microorganisms were inoculated into simulated wastewater at a 0.1% ratio to evaluate their pollutant degradation capacity by determining the degradation ratios of NH_4_^+^-N, TN, TP, NO_3_-N, and COD after a 24 h treatment at 30 °C and 150 r/min.

### Optimization of fermentation condition of PDBFs

Pollutant-degrading enzymes are induced enzymes. The composition and concentration of the culture medium affect the growth of microorganisms and their ability to reduce pollutants. However, the culture conditions mainly affect the growth of microorganisms. Therefore, the evaluation criteria were the concentration of viable bacteria and the ability to degrade NH_4_^+^-N to optimize culture medium components. The concentration of viable bacteria is the sole evaluation criterion to optimize culture conditions.

### Medium composition optimization

Based on the composition of HNLM, the effects of the carbon and nitrogen sources on the viable bacterial concentration and NH_4_^+^-N degradation ability were studied using a single-factor experiment. These factors were studied successively, and the optimized results were used in the subsequent steps. Sodium citrate, sodium succinate, sodium acetate, and glucose were used as alternative carbon sources. Beef paste, soybean meal, corn steep liquor powder, yeast extract powder, fish meal, peptone, tryptone, and urea were used as alternative sources of organic nitrogen. Ammonium sulfate and ammonium chloride were used as alternative sources of inorganic nitrogen. Based on the single-factor experiment, an Taguchi L9 (3^3^) orthogonal experiment was designed using SPSS software (version 20.0) to optimize the concentrations of the carbon and nitrogen sources.

Broths cultured in different media were centrifuged for 10 min at 5000 r/min. The precipitated PDBF cells were washed with PBS three times, inoculated into an inorganic salt medium at a concentration of 1.52 × 10^7^ cfu/mL, and cultured at 150 r/min and 30℃ for 24 h to evaluate their ability to degrade NH_4_^+^-N.

### Optimization of culture conditions

Based on the initial culture conditions (initial pH 7.0, culture temperature of 30 ℃, loading ratio of 40%, rotation speed of 150 r/min, inoculation ratio of 5%), the effects of temperature, pH, loading ratio, rotating speed, and inoculation ratio on the concentration of viable bacteria in culture broth were studied using a single factor experiment successively in a shake flask. All optimized results were used in the subsequent steps. Alternative temperatures were 20, 25, 30, 35, and 40 ℃. Alternative pH values of 5.0, 6.0, 7.0, and 8.0 were used. Alternative loading ratios of 2.5, 5.0, 10.0, 15.0, and 20.0% were used. Alternative rotating speeds of 90, 120, 150, 180, and 210 r/min were used. The alternative inoculation ratios were 1, 3, 5, 7, and 9%.

### Optimization of feeding strategy for fed-batch fermentation

Before optimization of the feeding strategy (Table 1) for fed-batch fermentation, a pre-test was conducted in a 10 L fermentation tank. The test condition were: loading ratio 50%, ventilation ratio 1:1 VVM, stir speed 300 r/min, tank pressure 0.05 MPa, and temperature 30 ℃. The optimum medium for flask culture (15.0 g/L sodium acetate, 0.9 g/L ammonium sulfate, 0.7 g/L soybean meal powder, 50.0 mL/L Vickers salt solution, and 10.0 mL/L trace elements) was adopted as the initial medium. The feed time was designed according to the growth curves of the screened PDBF (Additional file 1: Fig. S1).

**Table 1:** Feeding strategy

Batches	Content					Feed time (h)	Feed duration (h)
	Soybean meal (g)	(NH_4_)_2_SO_4_ (g)	CH_3_COONa 3H_2_O (g)	Vickers salt solution (ml)	Microelement (ml)		
1	1.75	2.25	37.5	125	25	15	3
2	1.75	2.25	37.5	125	25	15	5
3	0	2.25	37.5	125	25	15	3
4	0	1.125	37.5	125	25	15	3
5	0	6.75	37.5	125	25	15	3
6	0	11.25	37.5	125	25	15	3
7	0	9	37.5	125	25	15	3
8	0	6.75	37.5	125	25	12	3
9	0	6.75	37.5	125	25	15	6
10	0	0	0	0	0	0	0

^*^The Vickers salt solution contains 0.05 g/L FeSO_4_, 0.05 g/LMnSO_4_, 5.0 g/L K_2_HPO_4_, 2.5 g/L MgSO_4_ and 2.5 g/L NaCl

^*^Trace elements contains 5.5 g/L CaCl_2_, 5.06 g/L MnCl_2_, 1.1 g/L (NH_4_)_2_MoO_4_, 1.57 g/L CuSO_4_, 50.0 g/L EDTA, 2.2 g/L ZnSO4, 5.0 g/L FeSO_4_ and 1.61 g/L CoCl_2_

### Evaluation of the efficiency of PDBF in zeolite trickling filter towers

**Fig. 1:**
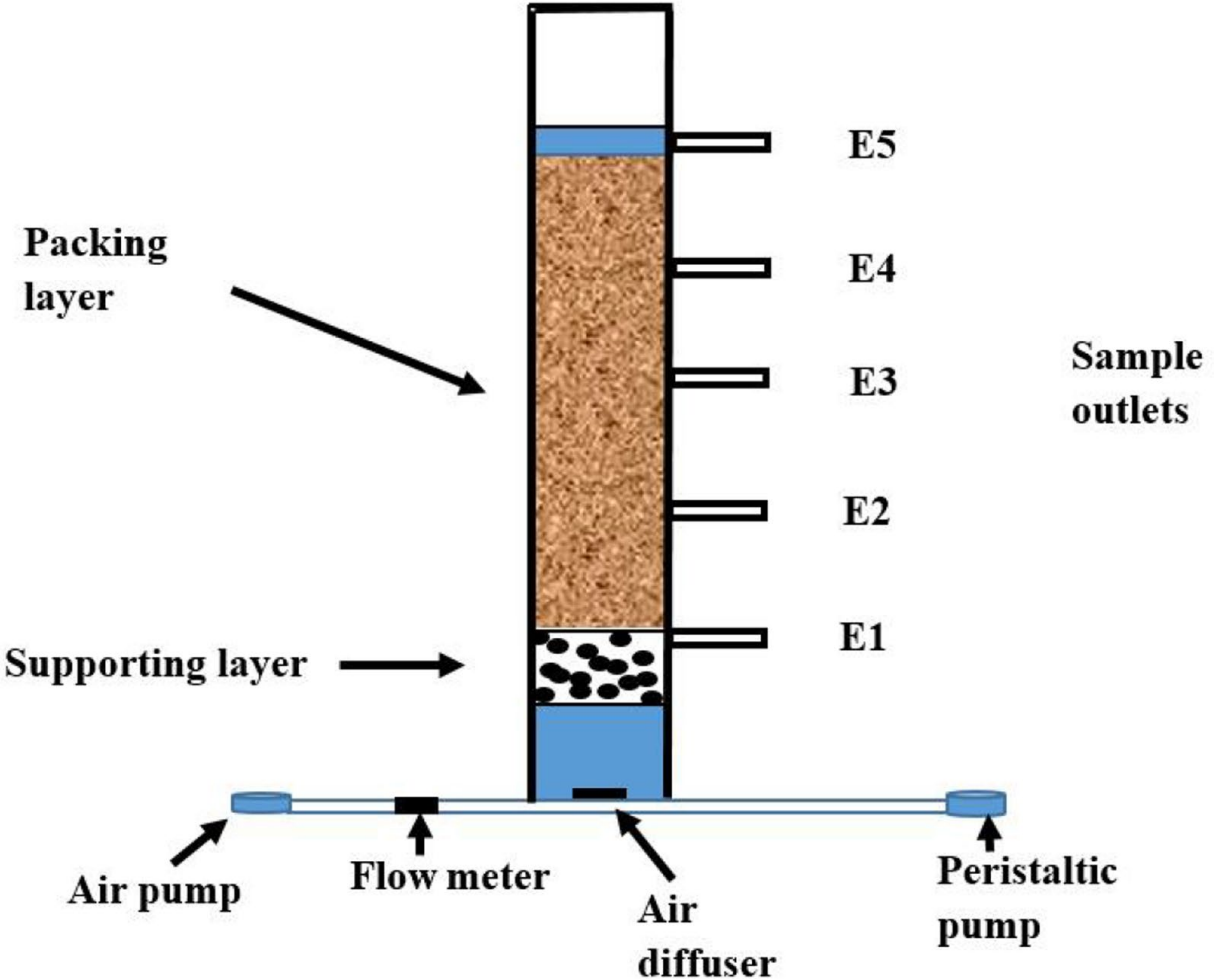
Schematic diagram of the experimental aerated zeolite trickling filter

The PDBF application verification experimental systems were constructed as 1200 mm × 150 mm diameter vertical Perspex tubes (Fig. 1), which were filled with zeolite (2–3 mm in diameter) as the main filter medium, with a 200 mm supporting layer filled with gravel (10–20 mm in diameter) at the bottom. There were five 150 mm interval sample outlets from the bottom to the top of the fillers and an air pump at the bottom of the system to create an aerobic environment. The experiments were performed in up-flow mode at 25 ℃.

Control and experimental groups were established. In the 10-d start-up procedure, a high-concentration nutrient solution (150 mg/L NH_4_^+^-N) mixed with mixed liquor volatile suspended solids (MLVSS, 3.723 g/L) was pumped into the system to stimulate microorganism growth and biofilm formation. The experimental conditions for the two groups were the same, except that the experimental group was inoculated with the studied microbial flora in addition to activated sludge. After the bacteria film was formed on the surface of the zeolite filler, simulated sewage containing 300.0 mg/L COD and 75.0 mg/L NH_4_^+^-N was pumped into the system to evaluate the pollutant degradation efficiency of the studied PDBF. The effluents were sampled from the sampling port E5 at 0 and 24 h to analyze the pollutant degradation ratio. The intermittent aeration mode was applied during the start-up and experimental processes, that is, aeration for 4 h to maintain the dissolved oxygen at 4–6 mg/L, then non-aerarion for 4 h. This process was repeated.

After the application effect evaluation experiment was completed, the biofilms on the surface of the zeolites located in E2 and E4 were soaked in sterilized saline solution. The microbial community composition was analyzed using second-generation sequencing.

## Analytical method

### Concentration of viable bacterial cells analysis

After sequential ten-fold dilution of culture broth, 100 µL of samples were spread on HNSM plates. Colonies formed after incubation at 30℃ for 16 h were counted and statistically analyzed. Viable counts were expressed as colony-forming units per milliliter (cfu/mL). Three replicates were used for each dilution.

### Pollutant degradation ratios analysis

The NH_4_^+^-N, TN, TP, and COD degradation rates were determined to evaluate the pollutant treatment efficiencies of the PDBFs. The concentrations of NH_4_^+^-N, NO_3_^−^-N, TN, TP, and COD were analyzed according to the China Standard Test Method for Water and Wastewater (China, 2002). The degradation rates of the pollutant indices were calculated as the ratio of the concentration of the treated sample to that of the untreated sample. Three parallel experiments were conducted for each experimental group.

### The microbial diversity sequencing method

The DNA of the biofilm obtained from the zeolite trickling filter was extracted using the PowerSoil DNA Kit (Mo Bio Laboratories, Carlsbad, CA, USA) according to the manufacturer's instructions. PCR amplification was performed using universal primers 341F (50′-CCTACGGGNGGCWGCAG-3′) and 806R (5′-GGAC-TACHVGGGTWTCTAAT-3′). Polymerase chain reaction (PCR) amplification was performed with Pfu polymerase using the following thermocycling program: denaturation at 95 °C for 5 min followed by 30 cycles of 95 °C for 30 s, 55 °C for 60 s, and 72 °C for 90 s, with a final incubation at 72 °C for 7 min. Purified PCR products were sequenced using the Illumina MiSeq platform (High-throughput Laboratory, Wuhan Huada Technology Service Co., Ltd.). The 16sRNAs of the samples (C, C1, C2, E1, E2) mentioned in the experiment have all been uploaded to the National Center for Biotechnology Information, and the associated BioProject accession numbers is PRJNA534376.

## Results

### Screening of PDBFs

The pollutant degradation parameters of the samples are listed in Table 2. Sample C exhibits the highest degradation rates of NH_4_^+^-N, NO_3_^−^-N, TN, and TP. Therefore, it was used in subsequent experiments.

**Table 2:** Pollutant degradation ability of candidate samples

Bacteria flora	Measurement parameters				
	NH_4_^+^-N (%)	NO_3_^−^-N (%)	TN (%)	TP (%)	COD (%)
A	79.9	17.4	56.7	58.4	40.9
B	43.3	11.5	28.99	9.7	0.0
C	82.3	50.4	78.95	66.3	62.6
D	0.9	3.0	75.4	38.5	95.2

### Optimization of fermentation technology of PDBFs

Optimizing the medium components and culture conditions of the microbial flora can improve the concentration of viable bacteria and pollutant degradation by liquid microbial agents, further promoting the degradation of pollutants in sewage. On the other hand, a high fermentation level is the key to reduce the preparation cost of solid sewage treatment microbial agents.

### Medium composition optimization

#### Effects of carbon and nitrogen sources on viable bacterial concentration and NH_4_^+^-N degradation rate of PDBF

The microbial flora grew well when the carbon sources were sodium acetate, citrate, or succinate (Additional file 1: Fig. S1a). Regarding their effect on the NH_4_^+^-N degradation rate, sodium succinate (81.5%) and sodium acetate (80.4%) were more effective than sodium citrate (77.2%). Considering both the viable bacterial concentration and the NH_4_^+^-N degradation rate, sodium acetate was used for further experiments.

According to Additional file 1: Fig. S1b, fish meal was the best nitrogen source, followed by tryptone and soybean meal. Nevertheless, the price of tryptone is much higher than that of fish meal and soybean meal powder (Li et al. 2022). Balanced by their effects and price, fish meal and soybean meal powder were used as candidate organic nitrogen sources. Additional file 1: Fig. S1c shows that ammonium sulfate is the best source of inorganic nitrogen for promoting PDBFs growth and NH_4_^+^-N degradation. Balanced by the effect on the screened PDBFs growth and increase in NH_4_^+^-N degradation ability, the mixture of 0.35 g/L soybean meal and 0.45 g/L ammonium sulfate was the best complex nitrogen source (Additional file 1: Fig. S1d.). As the optimization results show, carbon and nitrogen sources have a marked effect on the growth and pollutant-degradation ability of the flora. Suitable carbon and nitrogen sources positively affect the efficiency of microbial agents used in sewage treatment.

The concentrations of sodium acetate, ammonium sulfate, and soybean meal powder were optimized using an Taguchi L9 (3^3^) orthogonal experiment (Table 3). According to the range analysis results (Table 4), ammonium sulfate was the most important influencing factor for increasing bacteria cells concentration, followed by soybean meal; the optimal carbon and nitrogen source combination was 15.0 g/L sodium acetate, 0.9 g/L ammonium sulfate, and 0.7 g/L soybean meal powder. When cultured in this medium, the viable count in the broth reached 3.15 × 10^9^ cfu/mL.

**Table 3:** Orthogonal experimental factor level table

Level	Factor		
	A(sodium acetate) (g/L)	B(ammonium sulfate)(g/L)	C(soybean meal powder)(g/L)
1	5.00	0.45	0.35
2	10.00	0.90	0.70
3	15.00	1.35	1.05

**Table 4:** Orthogonal test results

Experiment No	A	B	C	Viable concentration (× 10^9^ cfu/mL)
1	3	3	1	0.63
2	1	2	3	1.01
3	3	1	3	1.12
4	1	3	2	0.74
5	2	3	3	1.21
6	3	2	2	3.15
7	2	2	1	1.05
8	2	1	2	1.11
9	1	1	1	0.83
K_1_	86	102	84	
K_2_	112	174	167	
K_3_	163	86	111	
R	77	88	83	

#### Optimization of culture conditions in shake flasks

**Fig. 2:**
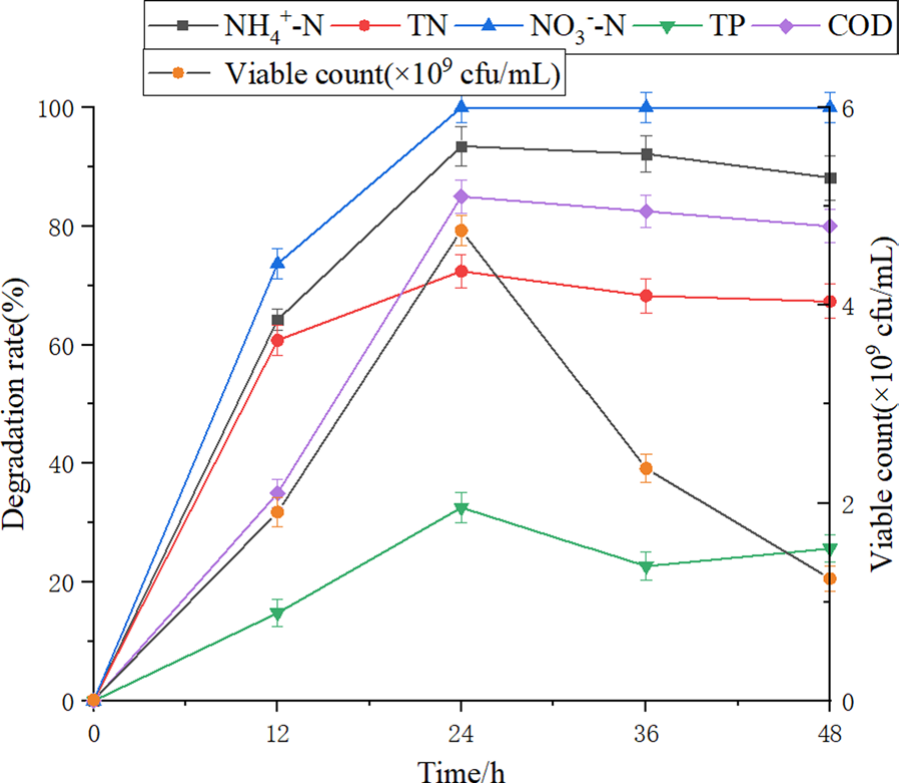
The viable concentration and pollutant degradation rates after optimization

As shown in Additional file 1: Fig. S2, the optimum culture conditions of the studied microbial flora were 30℃, pH 7, 10% loading ratio, 180 r/min rotating speed, and 3% inoculation ratio. Under these optimum culture conditions, the concentration of viable bacteria reached 3.19 × 10^9^ cfu/mL, 3.21 × 10^9^ cfu/mL, 4.55 × 10^9^ cfu/ml, 4.73 × 10^9^ cfu/ml, and 4.74 × 10^9^ cfu/mL, respectively. After culturing in the medium under all optimal conditions, the viable count of PDBFs reached 4.76 × 10^9^ cfu/mL. The degradation rates of NH_4_^+^-N, TN, TP, NO_3_^−^-N, and COD in simulated sewage were 93.5%, 68.3%, 32.6%, 100%, and 85%, respectively (Fig. 2), showing a significant increase compared to the results obtained under the initial conditions (Table 2).

#### Optimization of feeding strategy in 10 L fermentation tank

Based on the growth curves (Additional file 1: Fig. S3), the flora entered the logarithmic growth, stable, and decline phases at 3, 24, and 27 h, respectively. Nutrient supplements in the logarithmic growth phase extended this phase and increased the viable counts in the fermentation broth. According to the trial test results, feeding was started at 15 h.

**Fig. 3:**
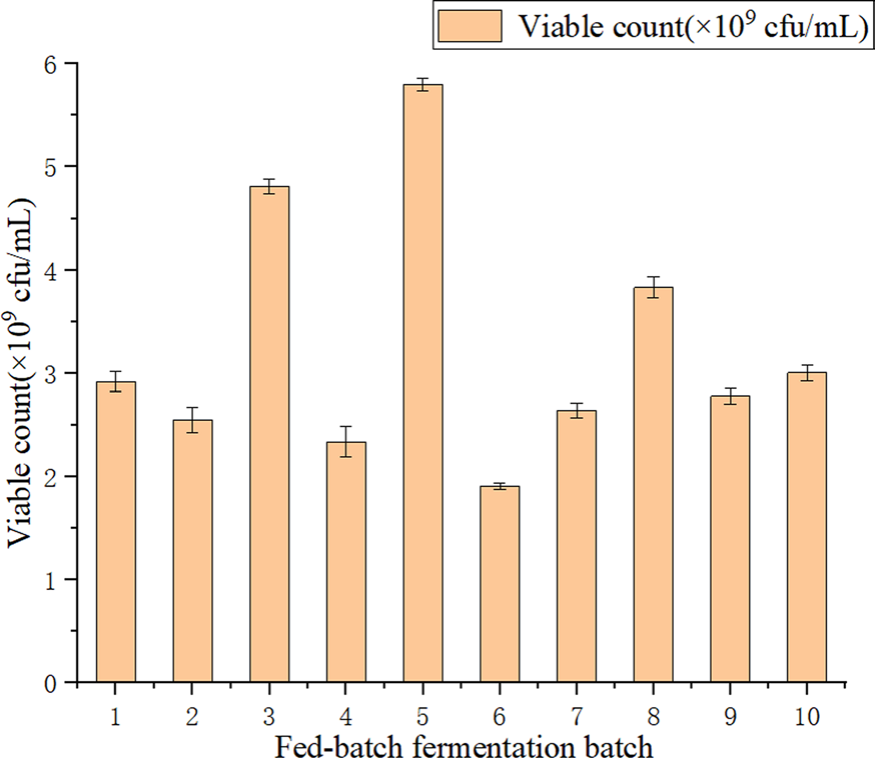
Effect of feed strategy on fermentation results in 10 Lfermentor (*1–9: different feed group as described in Table 1; *10: control group)

Ten batch experiments were conducted according to the feeding strategy (Table 1). Figure 3 shows that the fifth batch yielded the best results. The feeding composition was optimized as 37.5 g sodium acetate, 6.75 g ammonium sulfate, 125 mL Vickers salt solution, and 25 mL trace elements. The constant-speed feeding process started at the 15th hour and lasted for 3 h.

In the stationary phase, microorganisms began to produce pollutant-degrading enzymes, but their viable concentrations did not decrease. The late stable period was the optimal time to end fermentation; hence, the process was terminated at 36 h. After culturing for 36 h, the highest bacterial concentration reached 5.80 × 10^9^ cfu/mL, which was 1.91 times as high as that of the control experiment.

#### Evaluation of pollutant degradation efficiency of PDBFs in zeolite trickling filter towers

**Fig. 4:**
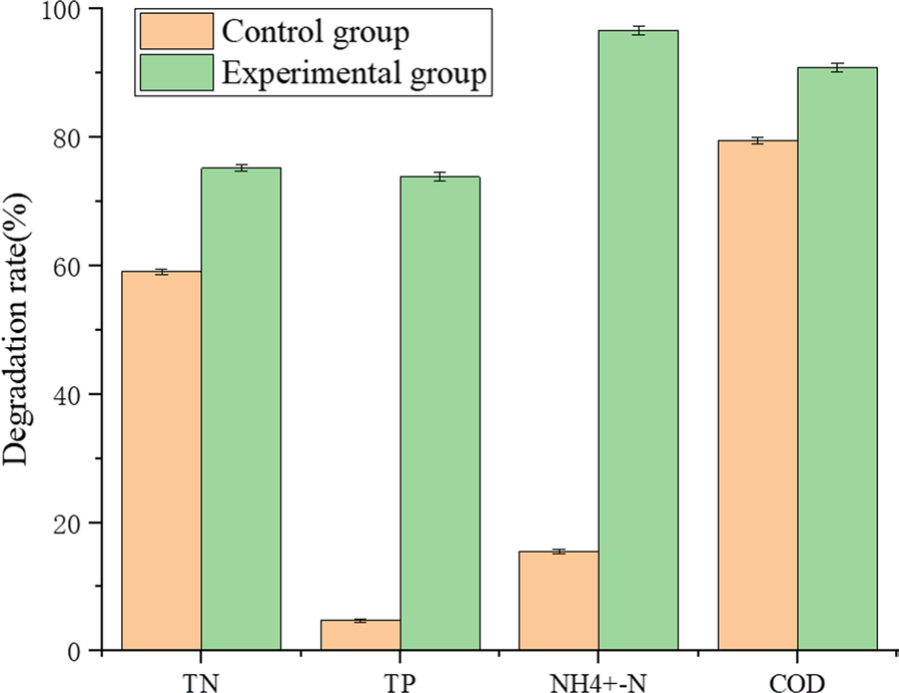
Comparison of pollutant degradation efficiency in ZTF systems

The pollutant degradation results of the control and experimental groups for the simulated wastewater after 24 h of treatment are shown in Fig. 4. The degradation rates of TN, TP, NH_4_^+^-N, and COD in the experimental group were 75.18%, 73.82%, 96.69%, and 90.83%, respectively, which were significantly higher than those in the control group (59.08%, 4.72%, 15.46%, and 79.44%, respectively). The improvements in the degradation effect of NH_4_^+^-N and TP were particularly significant.

#### The correlation between microbial community composition and sewage treatment efficiency

**Fig. 5:**
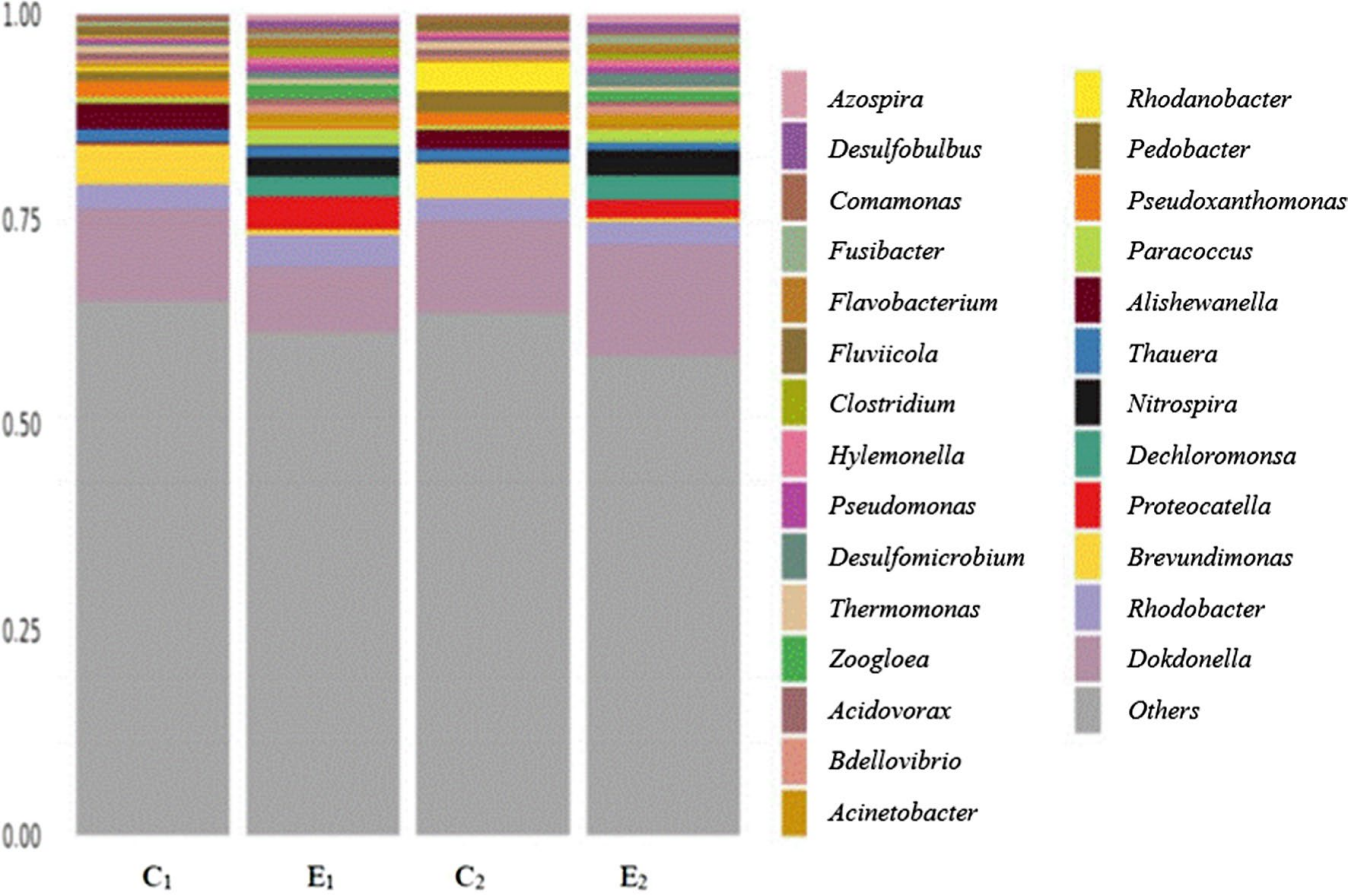
Histogram of species abundance at bacterial genus level (*The ordinate represents the relative abundance of species. C1 and E1 are the middle parts of the control group and theexperimental group respectively, and C2 and E2 are the upper parts of the control group and the experimental group respectively)

Figure 5 and Table 5 show that the microbiological components of sample C1 were similar to those of sample C2 but differed significantly from those of samples E1 and E2. The main microbial genera in samples C1 and C2 were *Dokdonella*, *Brevundimonas*, *Alishewanella*, *Rhodobacter*, *Pseudoxanthomonas*, and *Thauera*, while those of samples E1 and E2 were *Dokdonella*, *Proteocatella*, *Rhodobacter*, *Nitrospira,* and *Thauera*. *Proteocatella, Azospira,* and *Nitrospira* only existed in the biofilms of the experimental group. In addition to the above bacteria with high content, the experimental group also contained small amounts of *Acinetobacter*, *Acidovorax*, *Clostridium*, etc. (Fig. 5).

**Table 5:** Relative abundance of the samples at the generic level

OUT ID (genus)	Relative abundance (%)^a^			
	C_1_	C_2_	E_1_	E_2_
*Dokdonella*	33.33	30.87	20	33.96
*brevundimonas*	13.33	11.3	1.6	0.94
*Alishewanella*	8.89	6.52	0	0
*Rhodobacter*	6.67	9.78	10.2	5.47
*Pseudovanthomonas*	5.56	3.26	2.52	1.63
*Thauera*	4.44	4.35	4.57	2.16
*Proteocatella*	0	0	10.4	5.66
*Dechlomonas*	0	0	6	6.6
*Nitrospira*	0	0	5.2	7.55

^a^Others are not included in the calculation of relative abundance

## Discussion

Although microbial agents have recently been widely used for sewage treatment, most of them are expensive, and their pollutant removal efficiency is unstable. There is an urgent need to screen microbial agents with superior efficiency and reasonable price to meet market needs. Various pollutant-removing bacteria have been isolated, such as *Pseudomonas stutzeri* GEP-01 (Gao et al. 2020), *Arthrobacter nicotianae* D51st Rain (Cai et al. 2019), *Alcaligenes faecalis* strain WT14 (Chen et al. 2021), and phosphorus-solubilizing bacteria, such as *Pseudomonas moraviensis*, *Bacillus safensis*, and *Falsibacillus pallidus* (Wang et al. 2022). However, pure strains exhibit poor adaptability to complex sewage environments and cannot achieve satisfactory pollutant degradation. The strains in the composite flora can cooperate to improve their survival rate in the sewage environment, resulting in a higher pollutant degradation rate (Shen et al. 2017). In this study, the composite flora screened consisted of nitrifying bacteria, such as *Nitrospira* and *Rhodobacter*, and denitrifying bacteria, such as *Proteocatella*, *Dichloromonas,* and *Azospira*. A reasonable microbial community composition of the flora improved the pollutant-removal effect. When the simulated sewage was treated in a zeolite trickling filter tower,the degradation rates of NH_4_^+^-N, TN, TP, and COD reached 96.69%, 75.18%, 73.82%, and 90.83%, respectively.

A suitable culture environment can promote microbial growth (Li et al. 2022), and optimizing the culture environment is conducive to the removal of pollutants from sewage through microbial activities (Zhu et al. 2021). Research has shown that nitrifying or denitrifying bacteria must be present in a culture medium containing sufficient carbon sources to achieve a good nitrogen removal performance (Hu et al. 2021a). By optimizing the C/N ratio in the medium and the inoculation ratio of mixed bacteria comprising *Pseudomonas geniculata* ATCC 19374 and *Bacillus cereus* EC3, NH_4_^+^-N degradation rate increased from 92.84% to 99.84% and 78.6% to 96.9%, respectively (Liu et al. 2020). Li et al. reported that the concentration of viable bacteria in *Klebsiella* TN-10 culture medium was the highest when cultured at 30℃ with an initial medium pH value of 7, and the ammonia nitrogen degradation rate reached a peak of 96.4% after 24 h treatment under this condition (Li et al. 2019a, b). In this study, after optimizing the culture medium composition and culture environment of the microflora, the degradation rates of NH_4_^+^-N, TN, TP, NO_3_^−^-N, and COD reached 93.5%, 68.3%, 32.6%, 100%, and 85%, respectively, and the concentration of viable bacteria reached 4.76 × 10^9^ cfu/mL in the flask experiment.

Excessive nutrients in the initial medium cause the overgrowth of bacteria and rapid consumption of nutrients, leading to premature aging and autolysis of bacteria. Moreover, the growth of the flora is limited because of a lack of nutrition during the late fermentation stage. Proper feeding can prolong the logarithmic growth period, improve the final fermentation level, and achieve high-density fermentation (Scheel et al. 2021). After the feeding strategy was optimized in a 10 L fermentation tank, the highest bacterial cell concentration reached 5.80 × 10^9^ cfu/mL, which was 1.91 times as high as that of the control. In addition, compared with the growth of strains of single strain (*Rhodococcus* sp., *Pseudomonas* sp. et.) and mixed flora (*Pseudomonas geniculata* ATCC 19374 and *Bacillus cereus* EC3 Mixture), the number of viable bacteria is significantly higher.

Through analyzing the flora composition of the experimental and control group, we found that biofilms of the experimental group is rich in nitrifying and denitrifying bacteria. Among these, *Nitrospira* has good nitrification ability, and *Azospira* can use nitrogen in sewage (Daims and Wagner 2018). *Proteocatella* is a facultative anaerobic bacterium that decomposes urea in sewage which is also involved in the fossilization process of shrimp carcasses in water (Mahler et al. 2023), suggesting that it can reduce the pollutants produced by the decay of shrimp carcasses. *Dechloromonas* can realize simultaneous nitrogen and phosphorus removal in anoxic environments through denitrification and phosphorus removal metabolism, further reducing COD and aeration energy consumption in the sewage treatment process (Zhao et al. 2022). *Azospira* is a denitrifying bacterium commonly used for sewage treatment. Studies have shown that *Azospira* contains several denitrifying genes that can decrease the content of N_2_O; it is surmised that *Azospira* can reduce the content of TN and COD in sewage (Park et al. 2020). In addition to the above bacteria with high content, the experimental group also contained small amounts of *Acinetobacter*, *Acidovorax*, *Clostridium*, etc. (Fig. 5), which have nitrification or denitrification abilities. Their growth is promoted by enhancing the utilization of carbon sources and oxygen. (Zhu et al. 2017). This explains why the experimental group exhibited higher degradation rates of NH_4_^+^-N, TN, TP, and COD.

In conclusion, compound microbial flora with superior degradation efficiencies were screened. The composition of the medium and culture conditions were optimized to increase the concentration of viable bacteria. Under optimum culture conditions, the number of viable bacteria reached 4.76 × 10^9^ cfu/mL. The increase in the concentration of viable bacteria further increased the production of pollutant-degrading enzymes and the removal rate of pollutants, such as NH_4_^+^-N, TN, TP, and COD. After medium composition and culture condition optimization in the flask experiment, the degradation rates of NH_4_^+^-N, TN, TP, and COD reached 93.5%, 68.3%, 32.6%, and 85%, respectively, significantly higher than those before optimization. This study provides technical support for manufacturing microbial agents for sewage treatment with high pollutant-removal efficiency and reasonable cost. In addition, compared to single strains, composite floras have stronger adaptability and are more suitable for sewage treatment. This study provides an alternative microbial inoculum for sewage treatment.

### Supplementary Information

**Additional file 1: Figure S1.** Optimization results of medium composition. **a** Carbon sources: (1) sodium succinate (2) sodium citrate (3) sodium acetate (4) glucose (5) blank group; **b** Organic sources:(1) beef paste (2) soybean meal powder (3) Corn syrup dry powder (4) yeast extract powder (5) fish meal (6) peptone (7) urea (8) tryptone (9) blank group; **c** Inorganic nitrogen sources (1) 0.5 g/L ammonium sulfate; (2) 0.4 g/L ammonium chloride; (3) 1.0 g/L ammonium sulfate; (4) 0.8 g/L ammonium chloride; (5) blank group; **d** Complex nitrogen sources (1) ammonium sulfate 0.45 g/L + soybean meal powder 0.35g/L; (2) ammonium sulfate 0.45 g/L + fish meal 0.23g/L; (3) ammonium sulfate 0.5g/L; (4) ammonium sulfate 0.9 g/L + soybean meal powder 0.7g/L; (5) ammonium sulfate 0.9 g/L + fish meal 0.46g/L; (6) Ammonium sulfate is 1.00 g/L; **Figure S2.** Optimization results of culture conditions in shaking flask. **Figure S3.** The growth curve of the screened PDBF.

## Data Availability

All data generated or analyzed during this study are included in this published article and its Additional files.
