# Supplementary material for: A microbial flora with superior pollutant removal efficiency and its fermentation process optimization
Source: AMB Express. 2023 Oct 17;13:113. doi: 10.1186/s13568-023-01604-0 (PMC10581995; doi:10.1186/s13568-023-01604-0)
Supplement: Supplementary file 1 — Additional file 1: Figure S1. Optimization results of medium composition. a Carbon sources: (1) sodium succinate (2) sodium citrate (3) sodium acetate (4) glucose (5) blank group; b Organic sources:(1) beef paste (2) soybean meal powder (3) Corn syrup dry powder (4) yeast extract powder (5) fish meal (6) peptone (7) urea (8) tryptone (9) blank group; c Inorganic nitrogen sources (1) 0.5 g/L ammonium sulfate; (2) 0.4 g/L ammonium chloride; (3) 1.0 g/L ammonium sulfate; (4) 0.8 g/L ammonium chloride; (5) blank group; d Complex nitrogen sources (1) ammonium sulfate 0.45 g/L + soybean meal powder 0.35g/L; (2) ammonium sulfate 0.45 g/L + fish meal 0.23g/L; (3) ammonium sulfate 0.5g/L; (4) ammonium sulfate 0.9 g/L + soybean meal powder 0.7g/L; (5) ammonium sulfate 0.9 g/L + fish meal 0.46g/L; (6) Ammonium sulfate is 1.00 g/L; Figure S2. Optimization results of culture conditions in shaking flask. Figure S3. The growth curve of the screened PDBF. [file 13568_2023_1604_MOESM1_ESM.docx]

**a b**

**
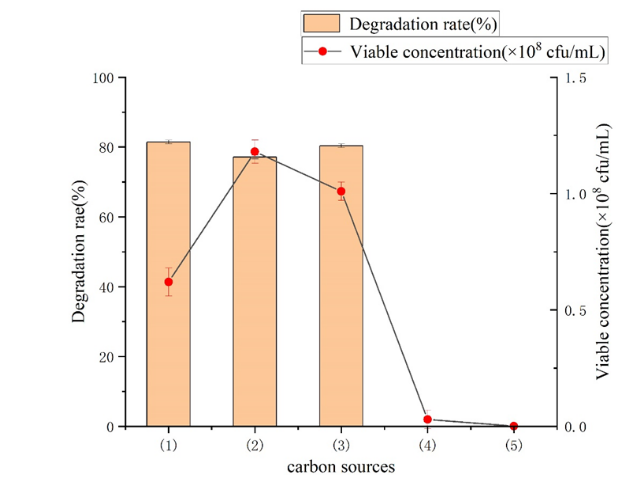

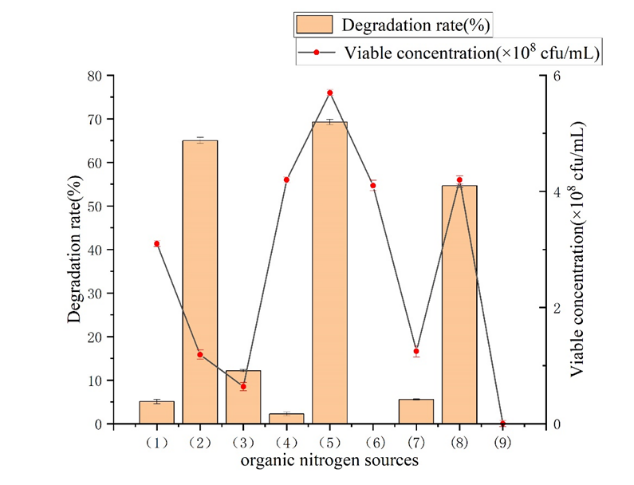
**

**c d**

**
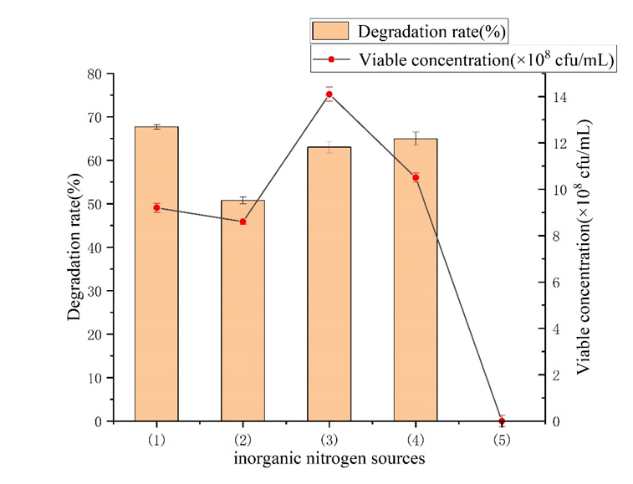

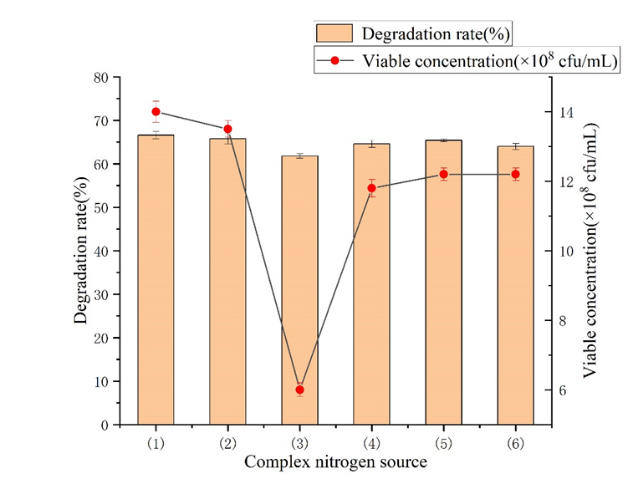
**

**a** Carbon sources: (1) sodium succinate (2) sodium citrate (3) sodium acetate (4) glucose (5) blank group;

**b** Organic sources :(1) beef paste (2) soybean meal powder (3) Corn syrup dry powder (4) yeast extract powder (5) fish meal (6) peptone (7) urea (8) tryptone (9) blank group;

**c** Inorganic nitrogen sources (1) 0.5 g/L ammonium sulfate; (2) 0.4 g/L ammonium chloride; (3) 1.0 g/L ammonium sulfate; (4) 0.8 g/L ammonium chloride; (5) blank group;

**d** Complex nitrogen sources (1) ammonium sulfate 0.45 g/L+ soybean meal powder 0.35g/L; (2) ammonium sulfate 0.45 g/L+ fish meal 0.23g/L; (3) ammonium sulfate 0.5g/L; (4) ammonium sulfate 0.9 g/L+ soybean meal powder 0.7g/L; (5) ammonium sulfate 0.9 g/L+ fish meal 0.46g/L; (6) Ammonium sulfate is 1.00 g/L;

**Fig. S1 Optimization results of medium composition**

**a** **b**


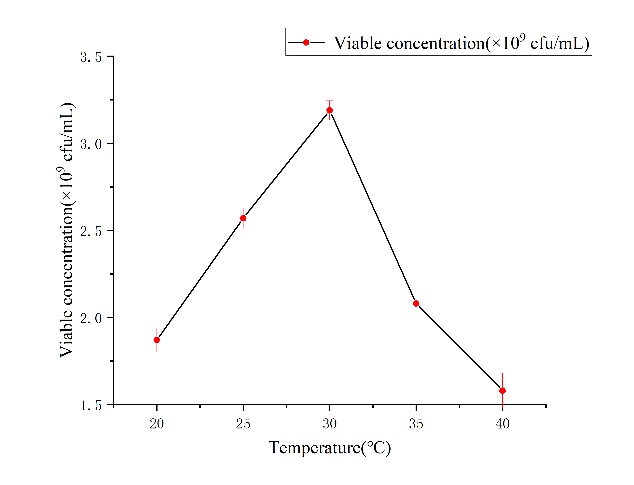

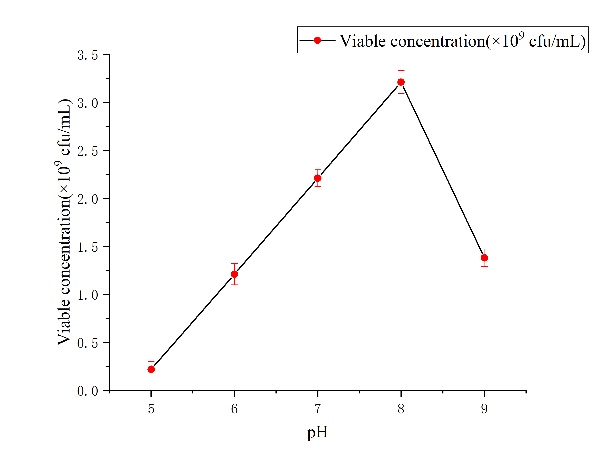


**c d**


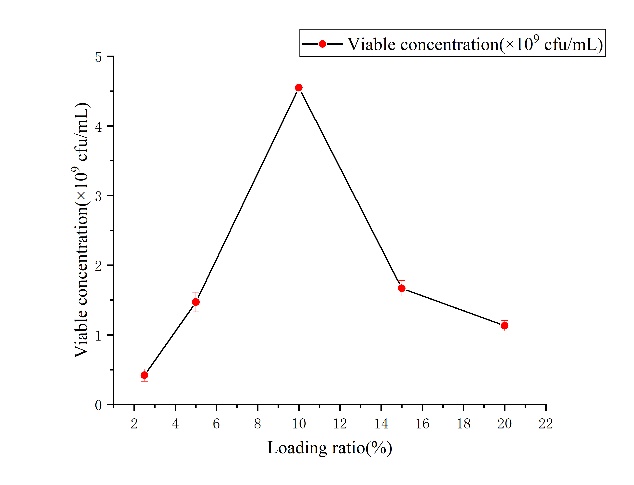

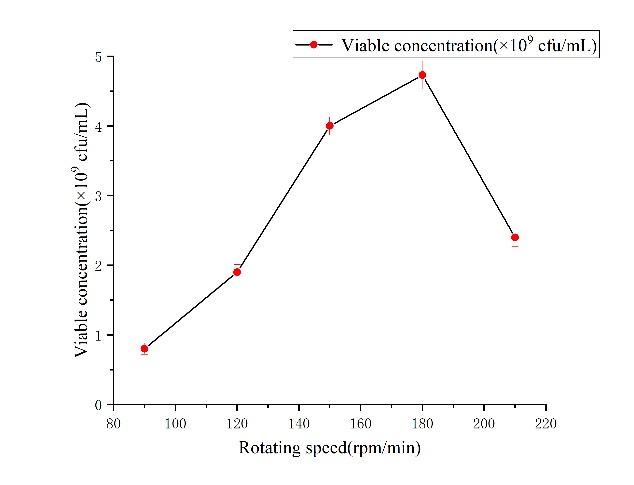


**e**


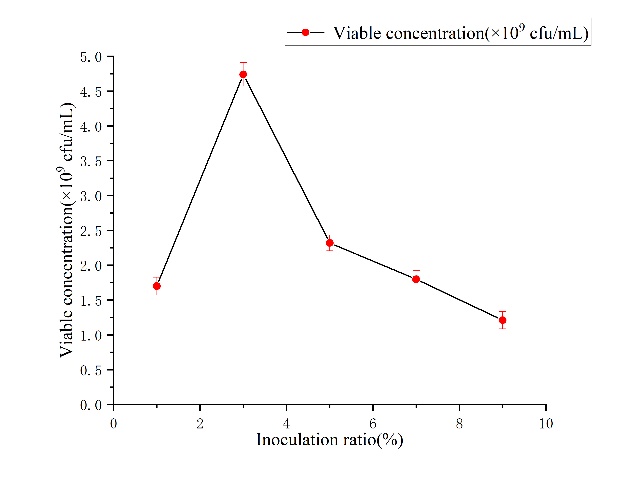


**Fig. S2 Optimization results of culture conditions in shaking flask**


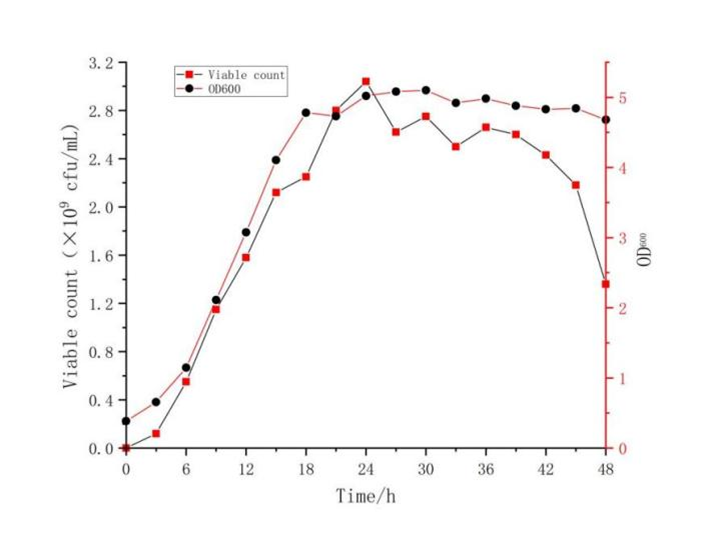


**Fig. S3 The growth curve of the screened PDBF**

**Table. S1 Price list of various carbon sources**

| Carbon sources | Price | | |
| --- | --- | --- | --- |
|  | **USD/Kg^a^** | **g/L^b^** | **USD/L^C^** |
| sodium succinate | 2.4 | 15 | 0.036 |
| sodium citrate | 0.67 | 15 | 0.01 |
| sodium acetate | 0.43 | 15 | 0.006 |
| glucose | 0.47 | 15 | 0.007 |

**^a^** Cost price of each component (accessed on December 2020)

**^b^** Component content per liter of culture medium

**^c^** Cost per liter of culture medium

* The special display represents the carbon source ultimately selected for this experiment

**Table. S2 Price list of various nitrogen sources**

| Nitrogen sources | Price | | |
| --- | --- | --- | --- |
|  | **USD/Kg^a^** | **g/L^b^** | **USD/L^C^** |
| beef paste | 6.24 | 0.7 | 0.004 |
| soybean meal powder | **0.56** | **0.7** | **0.0004** |
| Corn syrup dry powder | 0.53 | 0.7 | 0.0003 |
| yeast extract powder | 2.3 | 0.7 | 0.0016 |
| fish meal | 1.25 | 0.7 | 0.0009 |
| peptone | 3.36 | 0.7 | 0.0024 |
| urea | 0.38 | 0.7 | 0.0003 |
| tryptone | 4.61 | 0.7 | 0.003 |
| ammonium sulfate | 0.63 | 0.9 | 0.0006 |
| ammonium chloride | 0.67 | 0.9 | 0.0006 |

**^a^** Cost price of each component (accessed on December 2020)

**^b^** Component content per liter of culture medium

**^c^** Cost per liter of culture medium

*The special display represents the nitrogen source ultimately selected for this experiment
